# Mechanisms of Selenium Enrichment and Measurement in Brassicaceous Vegetables, and Their Application to Human Health

**DOI:** 10.3389/fpls.2017.01365

**Published:** 2017-08-03

**Authors:** Melanie Wiesner-Reinhold, Monika Schreiner, Susanne Baldermann, Dietmar Schwarz, Franziska S. Hanschen, Anna P. Kipp, Daryl D. Rowan, Kerry L. Bentley-Hewitt, Marian J. McKenzie

**Affiliations:** ^1^Plant Quality and Food Security, Leibniz Institute of Vegetable and Ornamental Crops Grossbeeren, Germany; ^2^Food Chemistry, Institute of Nutritional Science, University of Potsdam Nuthethal, Germany; ^3^Functional Plant Biology, Leibniz Institute of Vegetable and Ornamental Crop Grossbeeren, Germany; ^4^Department of Molecular Nutritional Physiology, Institute of Nutrition, Friedrich Schiller University Jena Jena, Germany; ^5^Food Innovation, The New Zealand Institute for Plant & Food Research Limited Palmerston North, New Zealand

**Keywords:** *Brassica* vegetables, selenium, biofortification, glucosinolates, human health, immune system, cancer, analytical methods

## Abstract

Selenium (Se) is an essential micronutrient for human health. Se deficiency affects hundreds of millions of people worldwide, particularly in developing countries, and there is increasing awareness that suboptimal supply of Se can also negatively affect human health. Selenium enters the diet primarily through the ingestion of plant and animal products. Although, plants are not dependent on Se they take it up from the soil through the sulphur (S) uptake and assimilation pathways. Therefore, geographic differences in the availability of soil Se and agricultural practices have a profound influence on the Se content of many foods, and there are increasing efforts to biofortify crop plants with Se. Plants from the Brassicales are of particular interest as they accumulate and synthesize Se into forms with additional health benefits, such as methylselenocysteine (MeSeCys). The Brassicaceae are also well-known to produce the glucosinolates; S-containing compounds with demonstrated human health value. Furthermore, the recent discovery of the selenoglucosinolates in the Brassicaceae raises questions regarding their potential bioefficacy. In this review we focus on Se uptake and metabolism in the Brassicaceae in the context of human health, particularly cancer prevention and immunity. We investigate the close relationship between Se and S metabolism in this plant family, with particular emphasis on the selenoglucosinolates, and consider the methodologies available for identifying and quantifying further novel Se-containing compounds in plants. Finally, we summarize the research of multiple groups investigating biofortification of the Brassicaceae and discuss which approaches might be most successful for supplying Se deficient populations in the future.

## Introduction

Awareness of malnutrition, e.g., deficiencies in iron, iodine, vitamin A, and zinc, in the developing world is high, but micronutrient deficiency is rarely discussed in developed countries. This reduced awareness is surprising, especially since micronutrient deficiency or suboptimal supply of the essential micronutrient selenium (Se) is seen in several developed countries such as New Zealand to (Thomson, 2004; Curtin et al., 2006), Australia (Oldfield, 2002) as well as in Europe, e.g., United Kingdom (Lyons et al., 2003), Germany (Hartfiel et al., 2010), and Finland (Alfthan et al., 2015), and is estimated affect about one billion persons worldwide (Haug et al., 2007). Micronutrient deficiency is usually regarded as having minor effects in developed countries where the diet is more diverse and the food comes from a range of sources rather than being limited to local produce. Therefore, Se deficiency in developed countries, such as New Zealand, is not as extreme as in the Se deficient areas of China and Tibet where local populations have suffered Se deficiency related disease that can be highly debilitating and sometimes fatal (Chen et al., 1980; Moreno-Reyes et al., 1998). However, there is increasing awareness that suboptimal amounts of Se can also be damaging to human health, in particular when coupled with malnutrition. Micronutrient deficiency may likely occur also in developed countries in the future, due to the consumption of fast-foods and the associated intake of so called “empty calories.”

As Se is an essential micronutrient, under-supply has direct and indirect consequences for human health. Direct disorders include a destabilized immune system, hypothyroidism and cardiomyopathy (Whanger, 2004; Rayman, 2012). Pathologic symptoms are developed as a consequence of a daily Se intake <10 μg day^−1^. Indirectly, Se deficiency results in loss of protective anti-cancerogenic effects through reduced expression of antioxidant selenoproteins and reduced availability of several seleno-compounds (Whanger, 2004). The dietary reference Se intake is 55 μg d^−1^ for adult humans in the USA, according to the National Institutes of Health^1^, whereas in Europe the recommended daily intake is 70 μg d^−1^ according to the European Food Safety Authority (EFSA Panel on Dietetic Products, Nutrition and Allergies (NDA), 2014).

Consequently, agricultural and horticultural food production systems should develop to improve access to more Se nutritious food. One promising approach is to promote the production and consumption of Se-biofortified plant-based food. It is remarkable that about 10% of the world's vegetable production is generated from Brassicales species (Augustine et al., 2014) including the most economically important family Brassicaceae. Brassicales species are able to accumulate Se (White, 2016) and, furthermore, are characterized by a certain group of secondary plant metabolites—the glucosinolates—found almost exclusively in the order Brassicales (Verkerk et al., 2009). Certain individual glucosinolates are known to confer health-promoting effects, mainly due to the anti-carcinogenic and antidiabetogenic properties of their hydrolysis products (e.g., Lippmann et al., 2014; Guzmán-Pérez et al., 2016). In contrast to other plant species, Brassicales species demonstrate the ability to synthesize not only seleno-amino acids and selenoproteins but also selenoglucosinolates. Moreover, synthetic breakdown products of selenoglucosinolates are reported to be distinctly more protective in cancer prevention compared to their S-containing analogs (Sharma et al., 2008; Emmert et al., 2010). It also seems likely that the antioxidant selenoproteins may be of benefit in counteracting diseases of oxidative stress such as cancer (Rayman et al., 2008). Previously several novel selenoglucosinolates have been identified from *Brassica* species (Matich et al., 2012, 2015; McKenzie et al., 2015b), and it was demonstrated that ingestion of Se-enriched broccoli, which contains these seleno-compounds alongside others, may have also a beneficial role in the human immune response (Bentley-Hewitt et al., 2014).

The focus of this review is to highlight the human health benefits implicit in the presence of unique Se-containing metabolites produced by *Brassica* species. This is an important and novel set of circumstances not present in other plant families—or contained in other reviews. The selenoglucosinolates in particular have not previously been reviewed in any depth, nor have their potential roles in human health. The review also highlights the latest methodology specific to the identification of Se containing compounds. Therefore, we aim to provide not only an up-to-date overview of previous and current research on Se metabolism in the Brassicales and its association with human health, but also provide new insight and motivation to further investigation.

## Selenium in brassicales

In recent years several reviews have been published on the importance of Se in higher plants, such as Terry et al. (2000), Pilon-Smits (2005), Zhu et al. (2009), Pilon-Smits and Quinn (2010), Feng et al. (2013), El-Ramady et al. (2015), Malagoli et al. (2015), Winkel et al. (2015), White (2016), and Schiavon and Pilon-Smits (2017). The review of El-Ramady et al. (2015) gives an overview regarding Se physiology and biology in higher plants and describes many aspects of Se fertilization, whereas Winkel et al. (2015) deals with Se uptake and pathways, but also with Se sources and distribution in water, air and soil. The groups round Terry and Pilon-Smits have established the critical nature of the selenocysteine methyltransferase (SMT) gene in the biosynthesis of methylselenocysteine (MeSeCys) and the role Se plays throughout the plants' wider ecosystem. White's (2016) latest review provides an excellent overview of Se uptake, translocation and metabolism in plants in general concluding in the demand to breed crops with greater Se concentrations in their edible tissue.

### Selenium uptake

As in most plant genera, the Brassicales take up Se primarily as the selenate anion (${\text{SeO}}_{4}^{2-}$), the predominant form occurring in alkaline and well-oxidized soils, as the selenite anion (${\text{SeO}}_{3}^{2-}$) existing in well-drained mineral soils, and also as selenocysteine (SeCys) and selenomethionine (SeMet; Ajwa et al., 1998).

Generally, the amount of Se taken up is related directly to the amount present in the soil (Brown and Shrift, 1982; Zhao et al., 2005) or the nutrient solution the plants grow on (Bañuelos, 1996). However, this is not always the case; for example in the *Brassica* vegetable rutabaga (*Brassica napus* L.) there was a poor correlation between Se uptake and Se soil content grown on a landfill (Arthur et al., 1992). Also, after two plantings of canola (*B. napus*), 80% of the Se remained in soils (Ajwa et al., 1998).

Se uptake has been shown to be greater when supplied in nutrient solution. While some experiments report data related to Se uptake from natural soils, most data originate from experiments in the context of biofortification (see Section Selenium Biofortification). The amount of Se taken up varies between species of Brassicales (Table 1). Concentrations may reach up to 2,000 μg/g dry weight (DW) (Ximenez-Embun et al., 2004; Manion et al., 2014). Significant genetic effects on Se concentration in Brassicales have been observed for leaves of rapid-cycling *B. oleracea* L. (Kopsell and Randle, 2001), broccoli florets [*B. oleracea* L. Italica Group (Bañuelos et al., 2003; Farnham et al., 2007; Ramos et al., 2011)], sprouts of cauliflower (*B. oleracea* L. Botrytis Group), kale (*B. oleracea* L. acephala Group), cabbage (*B. oleracea* Capitata Group) and Chinese cabbage [*B. rapa* L. (Ávila et al., 2014)], as well as shoots of Indian mustard [*B. juncea* (L.) Czern (Bañuelos et al., 1997)]. Comparing different *Brassica* species cultivated on natural soils with a comparable Se concentration of about 0.32 mg kg^−1^ the order of precedence in uptake (in μg g^−1^ DW) was Brussels sprouts (*B. oleracea* Gemnifera Group) (0.247), broccoli (0.129), savoy cabbage (*B. oleracea* Savoy Cabbage Group) (0.104), cauliflower (0.102), red cabbage (0.091), white cabbage (0.085), kale (0.046), kohlrabi (*B. oleracea* var. *gongylodes* L.) (0.037), and finally turnip (*B. rapa* var. *rapa* L.) (0.029; De Temmerman et al., 2014). This demonstrates the wide range of Se uptake within the Brassicales. As an example of the potential differences within a single species, cultivars of Indian mustard originating from different countries were compared under the same growing conditions. The amount of Se taken up doubled between the cultivar with the lowest and the highest Se concentrations independent of the supply form (soil or hydroponics) and the plant tissue (root or shoot; Bañuelos et al., 1997).

**Table 1:** Methods of Se enrichment in brassicaceous crops and resulting Se and MeSeCys content.

**Crop**	**Tissues analyzed**	**Se application method**	**Total Se in tissue μg g^−1^ DW**	**MeSeCys content μg g^−1^ DW**	**References**
Broccoli *Brassica oleracea* L. var. Italica Group	Florets	Hydroponic, mature plants, 20 μM selenite	1,200	<1.5 μmol	Lyi et al., 2005
	Florets	Soil fertilization, mature plants, 5 cultivars, 100 mL 1.5 mM Na_2_SeO_4_ 2x per week, 3 weeks	<558	<137	Ávila et al., 2013)
	Florets	Soil fertilization, mature plants, up to 5.2 mM selenate, every 2 days for 12 days (10 mL per plant for first 8 days, 20 mL per plant for last 4 days)	<879	nd^a^	Lee et al., 2005
	Florets Leaves	Greenhouse soil—non-saline irrigation, 250 μg Se L^−1^	<51 <31	nd^a^	Bañuelos et al., 2003
	Florets Leaves	Soil fertilization with increasing amounts dried Se-enriched *S. pinnata* (~700 μg Se g^−1^ DW), 23 weeks	<3.5 <3.5	7.4% soluble Se-compounds	Bañuelos et al., 2015)
	Florets	Soil enriched with *S. pinnata* (see Bañuelos et al., 2015) after 3 years	<8.0	5.0% soluble Se-compounds	Bañuelos et al., 2016
	Florets	Soil in pots enriched with up to 100 μM Na_2_SeO_4_ for up to 8 weeks	nd^a^	<3.4 μmol	Mahn, 2017
	Florets	Three field trials, SC, USA	<0.085	nd^a^	Farnham et al., 2007
	Sprouts	Hydroponic, sprouts, up to 100 μM Na_2_SeO_4_ or Na_2_SeO_3_ (1 week)	<263 (selenate) <185 (selenite)	<157 <167	Ávila et al., 2013
	Sprouts	Hydroponic, sprouts, 50 μM Na_2_SeO_3_ (1 week)	~180	~90	Ávila et al., 2014
	Sprouts	Hydroponic, 10 μg mL^−1^ selenite for 7 days	32 FW	94.3% of 0.2M HCl plant extract	Sugihara et al., 2004
	Sprouts	Hydroponic, 3 cultivars, 100 μmol L^−1^ Na_2_SeO_4_ or Na_2_SeO_3_ for 5 days	~85 (selenate) ~75 (selenite)	nd^a^	Tian et al., 2016
	Sprouts	Hydroponic, selenate 127/ 635/1270 μmol L^−1^	max. 100/120/245		Arscott and Goldman, 2012
	Leaves	Greenhouse soil non-saline irrigation, 250 μg Se L^−1^	<31		Bañuelos et al., 2003
	Leaves	Hydroponic, 20 lM Na_2_SeO_4_	<1,798		Ramos et al., 2011
	Head and upper stem	Foliar spray selenate, up to 20 mg Se plant^−1^ once, 3 month old plants ~2 mg Se plant^−1^, once, mature plants	55 5	nd^a^ nd^a^	Hsu et al., 2011
	Head, leaves, stem and roots in four cultivars	Foliar spray Na_2_SeO_4_, up to 50 g Se ha^−1^, once, mature plants	Up to 1,000 in head tissue, less in leaves, stems and roots	Up to 0.1 in head tissue	Sindelarova et al., 2015
	Shoot root	Weekly sand fertilization, young plants, 40 μM selenate for 6 weeks	420.7	nd^a^	Hsu et al., 2011
	Shoots	Hydroponic, seedlings, 38 broccoli accessions, 20 μM selenate for 2 weeks	<1,789	<0.8 FM	Ramos et al., 2011
	Stalks, roots Leaves, florets	Field trial, irrigated with drainage water 150 μg Se L^−1^	< 2.9 < 2.6 < 3.7 < 4.5		Bañuelos, 2002
Brussels Sprouts *B. oleracea* gemmifera Group	Sprouts	Hydroponic, sprouts, 50 μM Na_2_SeO_4_, 1 week	~50	~50	Ávila et al., 2014
Cabbage *B. oleracea* var. *capitata*	Sprouts	Hydroponic, 50μM Na_2_SeO_4_, 1 week	~180	~70	Ávila et al., 2014
	Leaves roots	Peat fertilization, up to 158 mg kg^−1^ peat as selenite:selenate (1:9), up to 6 months	1,606.793	nd^a^	Funes-Collado et al., 2013
	Leaves	Hydroponic: 2 mg L^−1^ Na_2_SeO_4_	120 max. 988 152 max. 531		Kopsell and Randle, 2001
Rapid cycling cabbage *B. oleracea* var. *capitata*	Shoots	Hydroponic, up to 9.0 mg L^−1^ selenate, 31 days	<732; < 1,740	nd^a^	Charron et al., 2001
	Leaf Stem root	Hydroponic, up to 9.0 mg L^−1^ selenate, young plants, 22 days	<1,916, <1,165 <1,636	nd^a^	Kopsell and Randle, 1999
	Leaf (seedlings)	Hydroponic, up to 1.5 mg L^−1^ Na_2_SeO_4_, 30 days	<375	nd^a^	Toler et al., 2007
Cauliflower *B. oleracea* var. *botrytis*	Sprouts	Hydroponic, sprouts, 50μM Na_2_SeO_4_, 1 week	~200	~90	Ávila et al., 2014
	Edible portion	Clay loam soil fertilization, up to 2.5 mg kg^−1^ soil as selenate	~30	nd^a^	Dhillon and Dhillon, 2009
Kale *Brassica oleracea* var. *sabellica* L.	Sprouts	Hydroponic, 50 μM Na_2_SeO_4_, 1 week	~180	~100	Ávila et al., 2014
	Seedlings	Hydroponic, up to 45 μg mL^−1^ Na_2_SeO_3_ <15 days	<386	<24	Maneetong et al., 2013
Turnip *B. rapa* ssp. *rapa*	Edible portion	Soil fertilization, up to 2.5 mg kg^−1^ soil as selenite	~60	nd^a^	Dhillon and Dhillon, 2009
	Sprouts	Hydroponic, 10 μg mL^−1^ selenite for 8 days	37 (FW)	94.5% of 0.2M HCl plant extract	Sugihara et al., 2004
Indian Mustard *Brassica juncea* (L.) Czern	Shoots/roots Shoots/roots	0.3-strength Hoagland solution + 4 mg L^−1^ Na_2_SeO_4_ Pot trial (soil/compost 7/3) 2 mg Se kg^−1^ substrate	<1,092/ <470 <769/ <332	0.006–0.215	Bañuelos et al., 1997
	Mature shoots	Se contaminated soils (5 μg g^−1^)	<60	nd^a^	Bañuelos et al., 2005
	Shoots roots	Hydroponics, max 15 mg L^−1^ Se, wild mustard	<1,300; <554	nd^a^	Bañuelos et al., 1990; Bañuelos, 1996
	Seeds	Plants grown on naturally Se-rich soil (6.5 mg Se kg^−1^ soil)	110 FW	nd^a^	Jaiswal et al., 2012
	Seeds	Sandy loam soil, three times weekly with 20 μM ${\text{SeO}}_{4}^{2-}$	<2.2	29% aqueous Se species	Bañuelos et al., 2012
	Leaves/stem	Soil loaden with 1.1 mg kg^−1^ total Se	<70/ <48		Bañuelos et al., 2000
	Shoot	Plants grown on naturally Se-rich soil (4.0 mg Se kg^−1^ soil), to 10 weeks old	~150	nd^a^	Van Huysen et al., 2004
	Seedlings	Hydroponic, up to 500 μM selenate, 1 week 150 μM selenite, 1 week	<200~400	5 FM nd^a^	Leduc et al., 2006
	Shoot /root Shoot /root	Hydroponic, seedlings, up to 5 mg L^−1^ Na_2_SeO_4_ or Na_2_SeO_3_, 2 weeks	2,081/3,411 58/605 (selenite)	nd^a^	Ximenez-Embun et al., 2004
	Shoot/roots	Hydroponic, 4 week old plants, up to 50 μM selenate for 8 days.	<1,800/ <960	nd^a^	Pilon-Smits et al., 1999
	Shoot/root	Hydroponic, 5 week old plants, 20 μM Se as selenate, or selenite, 1 week.	~500/~175 ~175/~35	nd^a^	Van Huysen et al., 2003
	Shoot/root	Hydroponic solution 20 μM Se as selenite, 1 week	~130/~145	nd^a^	De Souza et al., 1999
	Leaves/roots	Greenhouse, grown on seleniferous soil 1 mg kg^−1^	~125/~20	nd^a^	Cappa and Pilon-Smits, 2014
White mustard *Sinapis alba* L.	Seeds	Sandy loam soil, three times weekly with 20 μM ${\text{SeO}}_{4}^{2-}$	<1.3	17% aqueous Se species	Bañuelos et al., 2012
Canola (Oil seed rape) *Brassica napus*	Leaves Stem Roots	Soil fertilization, up to 1.5 mg kg^−1^, selenate and different organic forms	<284 <55 <88	nd^a^	Ajwa et al., 1998
	Leaves Stem Roots	Field trial, irrigated with drainage water 150 μg Se L^−1^	<6.2 <4.3 <3.1	nd^a^	Bañuelos, 2002
	Leaves/stem	Soil loaden with 1.1 mg kg^−1^ total Se	<80/ <30		Bañuelos et al., 2000
	Roots	Soil, 2 mg kg^−1^ total Se (${\text{SeO}}_{4}^{2-}$)	<315		Bañuelos et al., 1996
	Seeds	Sandy loam soil, three times weekly with 20 μM ${\text{SeO}}_{4}^{2-}$	< 1.7	20% aqueous Se species	Bañuelos et al., 2012
Radish *Raphanus sativus*	Edible portion	Soil fertilization, up to 2.5 mg kg^−1^ soil as selenate	~40	nd^a^	Dhillon and Dhillon, 2009
	Edible portion	Soils, containing 0.39 mg Se kg^−1^	~0.018		De Temmerman et al., 2014
	Seedlings	Hydroponic, selenite or selenium nanoparticles (1 mg L^−1^) for 40 days	207 144	47–72 Se species, 25–47 Se species	Palomo-Siguero et al., 2015
	Sprouts	Hydroponic, 10 μg mL^−1^ selenite for 8 days	21 μg g^−1^FM	96.5% of 0.2M HCl plant extract	Sugihara et al., 2004
Ethiopian kale *B. carinata* A.Braun	Shoot/root Shoot/root	0.3-strength Hoagland solution + 4 mg L^−1^ Na_2_SeO_4_ Pot trial (soil/compost 7/3) 2 mg Se kg^−1^ substrate	695/225 543/201	nd^a^	Bañuelos et al., 1997
Watercress *Nasturtium officinale* R.Br.	Shoot	Hydroponic, up to 4 mg Se L^−1^ as selenate, harvested at 28 d	Up to 2,550	nd^a^	Manion et al., 2014

a *nd, not determined*.

The form in which Se is provided is also important, with selenate being taken up two-fold faster than selenite in Indian mustard (De Souza et al., 1998). The order of preference when Indian mustard was supplied over an 8-day period with different Se forms at 20 μM was dimethylselenoproprionate (DMSeP) > SeMet > selenate > SeCys > selenite (Terry et al., 2000).

Although abiotic effects seem to be less influential than genetic effects, Se uptake also depends on environmental conditions and on the interaction with other nutrients supplied (see Section Promotion of Selenoglucosinolate Formation by Targeted Supply of N and S). In rapid-cycling *B. oleracea*, Se accumulation was clearly temperature-dependent (Chang and Randle, 2006), with Se concentrations increasing linearly with increasing temperature from 10 to 30°C in the leaves, ranging from 1.73 to 2.54 mg g^−1^ DW. Conversely, Se content decreased linearly with increasing temperature in the roots and ranged from 2.87 to 2.17 mg Se g^−1^ DW. Within environmental conditions, the microbiome also seems to be important for Se uptake. Inoculation of bacterial isolates into the rhizosphere of axenic plants (*B. juncea*) led to increased Se accumulation in shoots and roots following supply with 20 μmol selenate (De Souza et al., 1999). Depending on the strain selected, the tissue Se concentration increased up to three-fold in shoots and five-fold in roots. The influence of environmental factors has not yet been fully explored. However, knowledge in this area will become a crucial topic when Se-biofortification in crops is investigated.

Selenate enters root cells through sulphate transporters in their plasma membranes (Terry et al., 2000; White et al., 2004). sulphate transporters are encoded by a small family of genes; e.g., 14 in the genome of *Arabidopsis thaliana* L., and a similar number in other Brassicaceae species (Buchner et al., 2004; Hawkesford et al., 2005). A detailed overview of the sulphate transporters identified in *Arabidopsis* and their function are given in White (2016). Briefly, all sulphate transporters can be placed into one of four groups based on their protein sequences and distinct functional characteristics (Hawkesford, 2003; Hawkesford et al., 2005; Gigolashvili and Kopriva, 2014). Group 1 contains high-affinity sulphate transporters (HAST) that are thought to catalyse most selenate influx to cells (Hawkesford et al., 2005), sulphate transporters from group 2 are thought to catalyse selenate uptake into cells within the stele. Further, group 3 transporter AtSULTR3;5 appears to modulate the activity of a group 2 transporter, but does not catalyse transport itself (White, 2016). In contrast to group 1 transporters, sulphate transporters of group 4 (AtSULTR4;1 and AtSULTR4;2) might be responsible for catalysing the selenate efflux from the vacuoles (Gigolashvili and Kopriva, 2014). However, while these transporters are well-characterized in *Arabidopsis*, transporters and their mode of action need to be more fully investigated in brassicaceous vegetables.

For selenite uptake, P transporters are activated as well (Winkel et al., 2015). The involvement of the phosphate transport system in the movement of selenite throughout a plant has been reported based on the observation that increasing P concentration reduced selenite uptake rates in different plant species (Broyer et al., 1972; Hopper and Parker, 1999), however, this has not yet been found in Brassicales.

### Selenium mobilization and distribution

Selenate and selenite transport processes in all plants are energy-dependent (Hawkesford et al., 1993; Sors et al., 2005; Li et al., 2008). Selenate is rapidly translocated from the root to the shoot, whereas only ~10% of selenite is translocated in this way (De Souza et al., 1998). After uptake, Se is distributed within the plant to the different organs. The sites of accumulation depend on the species, its phase of development, and its physiological conditions. Overall, it follows: seeds > flowers > leaves > roots > stems (Terry et al., 2000; Quinn et al., 2011). A portion of the Se transported into the plant is volatilized as dimethyl selenide. Se-volatilisation rates of Indian mustard pre-treated for 7 d with 20 μg selenate amounted to 7 μg (g d)^−1^ DW and were two- to three-fold higher from plants pre-treated with 20 μg selenite (De Souza et al., 1999). A clear correlation was found between Se-volatilization rates and total Se concentrations.

### Selenium—a non-essential element for plants that has beneficial or toxic effects

Unlike in animals and some green algae (Araie and Shiraiwa, 2016), Se is considered a non-essential element for the healthy growth of crops (Zhang and Gladyshev, 2009). Brassicales as well as many other plant species exposed to high concentrations of Se in their root environment exhibit symptoms of injury. Visible and often initial symptoms are stunting of growth, root shortening, chlorosis, withering, and drying of leaves accompanied by decreased protein synthesis and ending in premature death of the plant (Terry et al., 2000). Toxicity thresholds are very different depending on the species and the environment.

In contrast, several authors report beneficial effects of increased Se content in the Brassicales, where low-dose Se supplementation has been shown to increase growth in *Stanleya* (Cappa et al., 2015), broccoli, radish (*Raphanus raphanistrum* ssp. *sativus* (L.) Domin), and turnip. This beneficial effect has been suggested to be due to Se-induced mimicry of S-deficiency resulting in increased S uptake by S transporters (Boldrin et al., 2016), increased anti-oxidant activity (Hartikainen et al., 2000; Proietti et al., 2013), and decreased lipid peroxidation (Xue et al., 2001; Abd Allah et al., 2016). Further, benefit of increased Se content derives from herbivory protection from insects, as shown in *S. pinnata, B. juncea* and *B. oleracea* Italica Group (Freeman et al., 2006a, 2007).

Based on their capacity for Se-uptake and tolerance plants are divided into three groups: Se non-accumulators, Se-indicators, and Se-accumulators (Brown and Shrift, 1982; Terry et al., 2000; White, 2016). The majority of plants are non-accumulating species, which cannot tolerate Se tissue concentrations of more than 10–100 μg g^−1^ DW, and rapidly show signs of Se toxicity (Hartikainen et al., 2001) on exposure to higher concentrations of Se than this. This toxicity is due to the non-specific incorporation of seleno-amino acids into proteins, replacing Cys and Met and thus disrupting protein function, and causing toxicity to the plant (Van Hoewyk, 2013).

Several members of the Brassicaceae fall into the category of Se indicator plants (also known as Se secondary accumulator plants) and are able to tolerate Se concentrations up to 1,000 μ g g^−1^ DW in their tissues and can therefore colonize soils described as seleniferous. These include broccoli (Lyi et al., 2005; Ramos et al., 2011; Ávila et al., 2013), Indian mustard (Bañuelos and Meek, 1989), kale (Maneetong et al., 2013), turnip, and headed cabbage (Sugihara et al., 2004; see Table 1).

Se-accumulator plants (also known as Se hyper-accumulators) are able to accumulate Se concentrations of >1,000 μg g^−1^ DW in their tissues with no apparent ill-effects (Pickering et al., 2003; Broadley et al., 2006; Freeman et al., 2006b; El Mehdawi and Pilon-Smits, 2012). Indeed, Se hyper-accumulators show a particularly strong growth effect which may exceed a two-fold increase in biomass production (El Mehdawi and Pilon-Smits, 2012). They are also the only plants able to colonize highly-seleniferous soils. Hyperaccumulation among the Brassicaceae family is found for *Cardamine hupingshanesis* (Yuan et al., 2013) and species within the genus *Stanleya* and *Thelypodium*, such as *S. pinnata* and *T. laciniatum* Endl. (Death et al., 1940; Galeas et al., 2007; Cappa and Pilon-Smits, 2014; Winkel et al., 2015). Although stems and leaves of the wildflower princesplume (*S. pinnata*) are edible and have been used as cooked greens and as medicine, when crushed they may have an unpleasant odor, are bitter and basically disliked (Whiting, 1985). It is intriguing that these species are able to accumulate an element that is not essential for higher plants (Zhang and Gladyshev, 2009), and that they not only tolerate but even grow better at tissue Se levels that are lethal for other plant species (Winkel et al., 2015).

### Metabolism of specialized selenocompounds in the brassicaceae

#### Methyl selenocysteine

Se-indicator or -accumulating Brassicaceae are able to take up and store excess Se due to the expression of an additional Se metabolism gene, SMT, which specifically methylates selenocysteine producing MeSeCys. MeSeCys is not incorporated into the plant's proteins, as SeCys or SeMet are, and therefore does not contribute to Se toxicity (Brown and Shrift, 1981). Instead it allows the safe storage of Se away from the plant's biosynthetic machinery. The SMT gene is inducible by selenate in broccoli (Lyi et al., 2005) and has been confirmed as the key to Se-tolerance in Se-accumulating plants (Neuhierl and Bock, 1996; Neuhierl et al., 1999). Its over-expression in non-Se accumulator species, such as tobacco and tomato, has been shown to convert such plants into Se-accumulators with up to 25% of the Se in these plants found as MeSeCys (McKenzie et al., 2009; Brummell et al., 2011).

MeSeCys content has been reported for many brassicaceous crops fertilized with Se (Table 1). Ávila et al. (2014) hydroponically fertilized sprouts from six different *Brassica* species with 50 mM selenate for 1 week and reported MeSeCys concentrations of up to 50–100 μg g^−1^ DW (Table 1). Sugihara et al. (2004) have also reported high quantities of Se as MeSeCys in broccoli, Chinese cabbage, radish, and turnip sprouts. There have been few reports of MeSeCys concentration in mature crop plants, though 108 μg g^−1^ DW (Ávila et al., 2013), 1.5 μmol g^−1^ DW (Lyi et al., 2005), and 3.4 μmol g^−1^ DW (Mahn, 2017) have been reported in broccoli florets. MeSeCys and its derivative γ-glutamyl methylselenocysteine, have been shown to have greater bioefficacy in preventing cancer cell proliferation than other Se-containing compounds (Whanger, 2002). These compounds are also believed to be responsible for the reported decreased rate of pre-cancerous cell production in rat models following ingestion of Se-enriched broccoli (Finley et al., 2001). Thus, the presence of MeSeCys is important when considering Se-metabolism in the Brassicales in the context of human health.

#### Selenoglucosinolates

**Figure 1:**
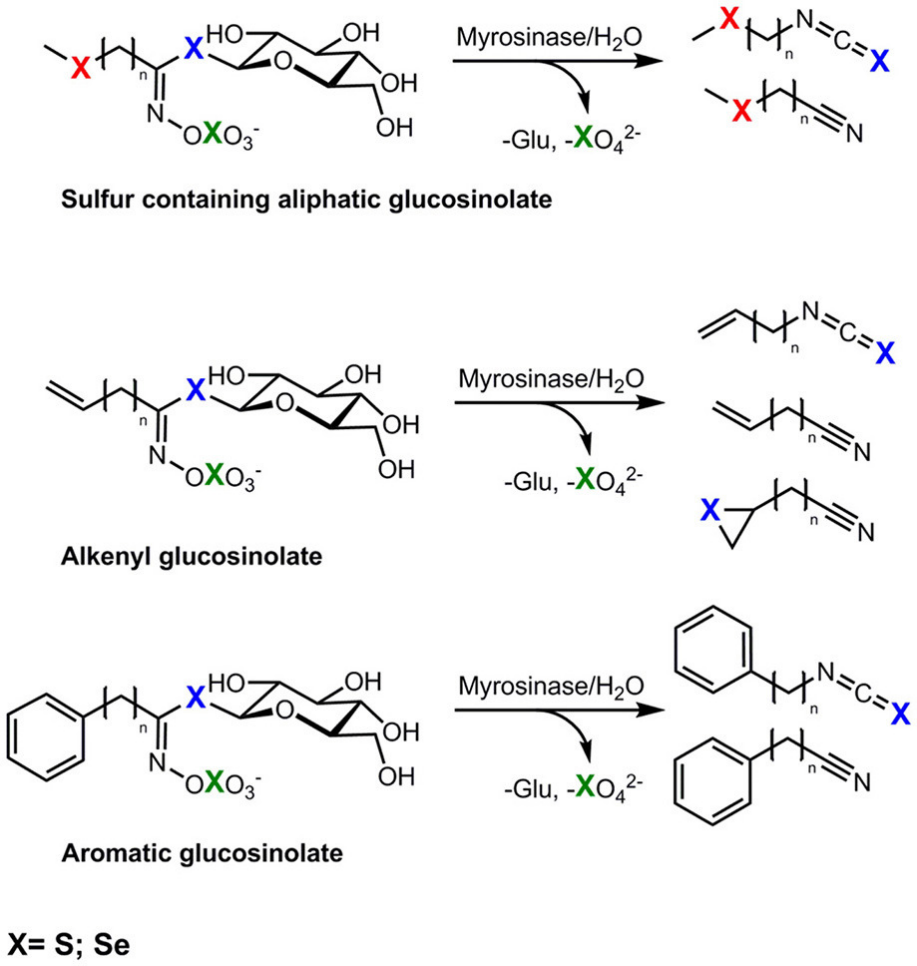
Possible exchange positions (X) of sulphur (S) by selenium (Se) in seleno-glucosinolates and the formation of possible corresponding hydrolysis products, red, incorporation in side chain; blue, incorporation in thioglucose, green, incorporation in sulphate group.

Selenium *Brassica* accumulators contain glucosinolates, a group of secondary plant metabolites containing sulphur. Glucosinolates are β-d-thioglucoside-*N*-hydroxysulphates with a variable side chain. So far more than 130 glucosinolates have been reported (Agerbirk and Olsen, 2012). Due to their variable side chain glucosinolates can be classified into aliphatic, aromatic, or indole forms. The aliphatic glucosinolates can be subdivided into straight or branched chain aliphatics, alcohols or unsaturated alkenyl glucosinolates, as well into the sulphur containing aliphatic methylsulphanylalkyl (SII), methylsulphinylalkyl (SIV), or methylsulphonylalkyl glucosinolates (SVI; Hanschen et al., 2014). Thus, according to their structure, glucosinolates contain at least two, very often three, and sometimes four sulphur atoms that might be replaced by Se in plants grown in Se-rich soils. Upon cell disruption, glucosinolates are hydrolyzed by the endogenous plant enzyme myrosinase, resulting in the formation of volatile hydrolysis products such as nitriles and isothiocyanates (Kissen et al., 2009). Possible breakdown products are shown in Figure 1. Isothiocyanates are valued as pleiotropic agents that exert a multitude of cancer-preventive actions, among them chemopreventive phase-I enzyme inhibition and phase-II enzyme induction as well the induction of apoptosis and cell cycle arrest. Thus, these compounds are linked to the cancer-preventive effects of *Brassica* consumption (Veeranki et al., 2015).

As glucosinolates are precursors to cancer preventing substances and exchanging sulphur with Se might further enhance the bioactivity of glucosinolate hydrolysis products (Emmert et al., 2010), it is of great interest to study the effect of Se on glucosinolate production and the formation of selenoglucosinolates.

More than 40 years ago Stewart et al. (1974) reported a Se-containing sinigrin (2-propenyl glucosinolate) in horseradish (*Armoracia lapathifolia* Gilib.). In 1988, Kjær and Skrydstrup (1987) synthesized the first Se-containing glucosinolates by replacing the thioglucosidic S with Se in order to study their properties and their enzymatic hydrolysis. One year later, their group identified traces of selenogluconapin (Se-3-butenyl glucosinolate) and the corresponding isoselenocyanates in plants of *S. pinnata* after 3-weeks of Se fertilization with 100 ppm sodium selenite (Bertelsen et al., 1988), indicating incorporation of Se into the glucose moiety. However, the ratio of Se-glucosinolate to the normal glucosinolate did not exceed 1:50,000. Further, the authors did not detect significant Se incorporation in *Lepidium sativum* L., *A. lapathifolia* nor in *S. pinnata* grown at low Se-levels (Bertelsen et al., 1988). Higher incorporation of Se into glucosinolates of *Brassica* species was reported by Matich and co-workers in 2012. Following treatment with sodium selenate (20 mL of 5 mM, twice weekly to the soil for 4 weeks) to forage rape (*cv*. Maxima), cauliflower (*cv*. Liberty), and broccoli (*cv*. Triathlon) the accumulation of methylselenoalkyl glucosinolates with up to 40% of the respective glucosinolate containing Se was reported (Matich et al., 2012). Further, they analyzed the respective volatile hydrolysis products and identified methylselenoalkyl nitriles and isothiocyanates indicating that Se was incorporated into the methylselenoalkyl side chain of the glucosinolate (Matich et al., 2012). For example, the main isothiocyanate, the 4-methylselenobutyl isothiocyanate was found in 3-times higher concentrations compared to the sulphur analog (Matich et al., 2012). In 2015, that group went on to study the distribution of Se-glucosinolates and their metabolites in these plants and reported that broccoli florets particularly accumulate methylselenoalkyl glucosinolates with up to 32.4 μg g^−1^ FW and about 50% of the glucosinolates present being selenised (Matich et al., 2015). Moreover, incorporation of Se into aromatic glucosinolates such as gluconasturtiin (2-phenylethyl glucosinolate) was observed. However, this was at very low rates (only 0.04% of the glucosinolate) and the authors observed no isoselenocyanate formation (Matich et al., 2015). Thus, Matich and coworkers concluded that selenoglucosinolate biosynthesis in *Brassica* via SeMet is the only efficient route (Matich et al., 2012, 2015).

Recently, Ouerdane et al. (2013) tentatively identified several selenoglucosinolates in seeds of *B. nigra*. As well as methylselenoalkyl glucosinolates such as glucoselenoerucin or glucoselenoiberin [4-(methylseleno)butyl- and 3-(methylseleninyl)propyl glucosinolate], they reported the detection of several non-typical glucosinolates. They postulated methylseleno-indole glucosinolates and glucosinolates acylated with methylselenoacetic acid or with methylselenosinapinic acid at position 6' of the thioglucose moiety. Typically, acylated glucosinolates are only found in seeds and not in other plant parts (Agerbirk and Olsen, 2012).

#### Promotion of selenoglucosinolate formation by targeted supply of N and S

The amounts of N- and S-containing compounds in plants, such as glucosinolates, can be highly variable and are strongly influenced by S and N supply (e.g., Kim et al., 2002; Li et al., 2007; Schonhof et al., 2007). Thus, *Brassica* species-specific N/S ratios distinctly stimulate the formation of glucosinolates (Fallovo et al., 2011). For example, N/S ratios between 7:1 and 10:1 promoted aliphatic alkyl and indole glucosinolates concentrations in broccoli florets (Schonhof et al., 2007), whereas high concentrations of aromatic glucosinolates occurred in turnip roots at N/S ratios <5 (Li et al., 2007). It seems that within these ranges of N/S the corresponding glucosinolate precursors of the amino acid-derived glucosinolates would be preferentially available for glucosinolate synthesis (and not for protein synthesis), such as Met for aliphatic glucosinolates and phenylalanine and tryptophan for aromatic and indole glucosinolates, respectively. Matich et al. (2015) suggested that SeMet is the decisive Se-precursor bottleneck or the major Se-precursor for the formation of selenoglucosinolates. Consequently, a targeted strategy for selenoglucosinolate production in *Brassica* plants could include the identification of a N/S/Se ratio and how to balance it for the promotion of selenoglucosinolate synthesis.

### Selenium biofortification

As Se is lacking in many diets, consumption of plants containing Se may be an effective way to increase dietary Se (McKenzie et al., 2015a). Furthermore, Se enrichment of the Brassicales produces Se-containing compounds with added bioefficacy, such as MeSeCys and potentially the selenoglucosinolates. To reach RDIs of >55 μg d^−1^ as recommend for adult humans a 100 g serving of fresh *Brassica* food with a Se concentration of at least 5 μg g^−1^ DW is necessary. This can be achieved via biofortification; the idea of enhancing nutrients in food crops. Three methods of Se-biofortification have been used for the Brassicales; hydroponic culture, soil fertilization and foliar spraying, resulting in varying amounts of Se uptake (for references, see Table 1).

The highest reported Se concentration in the Brassicaceae have been recorded following hydroponic culture (Table 1), with Se concentrations of 1,200 and up to 1,800 μg Se g^−1^ DW reported in the florets and leaves of broccoli (Lyi et al., 2005; Ramos et al., 2011), up to 1,900 μg Se g^−1^ DW in rapid cycling cabbage leaves (Kopsell and Randle, 1999), and up to 1,800 and 2,000 μg Se g^−1^ DW in *B. juncea* shoots and seedlings, respectively (Bañuelos et al., 1990; Pilon-Smits et al., 1999; Ximenez-Embun et al., 2004). Presumably, the constant exposure of the plant's root system to the Se-enriched solution and the lack of Se-soil interactions act together to make hydroponic culture particularly efficient. The Se concentrations achieved are dependent on the concentration of Se in the hydroponic solution and the length of time the plant is exposed, as well as the Se-accumulating capacity of the plant species, therefore direct comparisons between different experiments are difficult. However, broccoli, Indian mustard, and rapid-cycling *B. oleracea* appear to accumulate the highest Se contents following hydroponic culture (1,800–1,900 μg g^−1^ DW in the shoot tissue, Table 1), taking them into the realm of the Se-hyper-accumulators. By comparison, the hydroponic culture of Brussels sprouts, cabbage, cauliflower, Chinese cabbage, kale, radish, and turnip for similar lengths of time and Se concentrations result in lower Se accumulation (50–386 μg g^−1^ DW; Table 1).

Soil fertilization has also been used for Se enrichment, particularly for broccoli, resulting in a maximum reported content of 879 μg g^−1^ DW in the florets of mature, flowering plants fertilized with Se every second day for 12 days (Lee et al., 2005; Table 1). Recently, material from the Se-hyper-accumulator *S. pinnata* that had been grown on seleniferous soils was used to enrich the Se content of the soil broccoli plants were subsequently grown in. This resulted in a Se content of 3.5 μg g^−1^ DW in the leaves and floret material of the broccoli (Bañuelos et al., 2015). Indian mustard plants grown on naturally Se-rich soils have been shown to accumulate Se up to 150 μg g^−1^ DW in their shoot material (Van Huysen et al., 2003). Soil fertilization of other brassicaceous crops resulted in Se contents of 30–60 μg g^−1^ DW in cauliflower, radish and turnip (Table 1). Notably, when cabbage was grown on peat fertilized with Se at 158 mg kg^−1^ soil for 6 months Se accumulated to 1,600 μg g^−1^ DW in the leaves with no toxicity symptoms (Funes-Collado et al., 2013). This is a high concentration and presumably due to the length of time the plants were exposed to the Se treatment.

In order to develop a commercial regime for Se-enrichment of broccoli, Hsu et al. (2011) investigated foliar application of sodium selenate as a single dose to the leaves of plants growing in the field, resulting in head and upper stem tissue containing 5 μg g^−1^ DW Se. Recently, Palomo-Siguero et al. (2015) investigated the bioefficiency of Se supplied as Se-nanoparticles to the roots of radish plants hydroponically. There was no sign of Se toxicity in plants treated this way and the Se were incorporated into MeSeCys and SeMet. Se accumulation was 25% less when Se nanoparticles were used compared with selenite (Table 1). Nevertheless, this is the first report of Se being biotransformed from nanoparticles in plants.

Sodium selenate and sodium selenite are the most commonly used Se-sources for the enrichment of *Brassicas*. However, plants exposed to selenite have a much reduced Se content compared to those fertilized with selenate (Ximenez-Embun et al., 2004; Lyi et al., 2005). This is because selenate is taken up directly by high efficiency S transporters in the roots compared with the less efficient phosphate transporters used to take up selenite (see Section Selenium Uptake).

Transgenic approaches have also been used to successfully increase the Se and MeSeCys content of *Brassica* species. Over-expression of ATP-sulphurylase, the rate limiting step for Se uptake and assimilation, in *B. juncea* resulted in a two- to three-fold increase in Se content in the shoots (Pilon-Smits et al., 1999). A similar approach was used for SMT in *B. juncea*, resulting in up to 4,000 Se μg g^−1^ DW accumulating in seedlings, and 100 μg Se g^−1^ FW as MeSeCys; a four-fold increase compared with controls (Leduc et al., 2004). However, although effective, a transgenic approach to increasing Se content in crops is unlikely to be acceptable by consumers in the near future. Gene editing technologies such as CRISPR/Cas9, may offer an alternative, and ultimately more palatable method, though this technology is not so easily applied to the upregulation of genes as to down.

When producing vegetables with enhanced Se concentrations it is necessary to consider that toxic conditions might be reached in the diet of some consumer groups such as children or people with particularly high vegetable consumption. Therefore, it is important to produce plants with stable and defined Se contents so that food produced from these are safe and where advice on quantities for consumption can be relied upon. Careful consideration should be given to the amount of Se taken up by different Brassicales as well as reproducibility over the growing season. The amount of Se taken up can most effectively be controlled under hydroponic conditions. However, it is important to note that even using this method substantial differences have been noted for Se uptake in the same species (Table 1). Therefore, the amount of Se applied to a *Brassica* crop should be carefully determined in each case and over several growing seasons. Despite this, biofortification of crops through Se-enriched fertilizers has been conducted in Finland for the past two decades as the population was Se-deficient. Currently, the daily Se intake for the Finish population is considered to meet the Nordic and EU RDI (Alfthan et al., 2011). Thus, understanding mechanisms of Se-uptake into crop plants and how this is affected by environmental conditions is of great importance in producing biofortified plant foods.

## Instrumental approaches for detecting, measuring, and monitoring selenium and its metabolites within *Brassica* species

Selenium readily substitutes for S in a non-specific manner in biological systems and is thus readily incorporated by living organisms into a variety of organic compounds which would normally contain sulphur. This process results in the biosynthesis of Se-containing amino acids, proteins and plant secondary metabolites such as glucosinolates. Biogenic production of methylselenol (MeSeH) from selenoamino acids may also result in the production of further Se-containing metabolites including selenosugars, selenosinapine, and selenourea derivatives as shown in mustard seeds (Ouerdane et al., 2013). We briefly summarize instrumental approaches for detecting, measuring, and monitoring Se and Se-containing metabolites.

### Total and inorganic selenium

Inductively coupled plasma-optical emission spectrometry (ICP-OES) provides quantification by measuring the light emitted from excited ions and atoms at characteristic wavelengths. Excited ions and atoms are formed by reaction of the sample in an inductively-coupled plasma (ICP) and detected by atomic emission. For Se, the detection limit is relatively high due to the poor emission intensity of Se compared to other elements. The primary detection wavelength is 196.026 nm with small interference with iron complicating the analysis of Se in iron rich samples (Ralston et al., 2008). ICP can be also coupled to mass analysers, typically a quadrupole mass spectrometer (MS) in ICP-MS. In addition, high resolution ICP-MS systems can be used to resolve Se-isotopes. Important in ICP-MS is the consideration of potential interferences and to minimize biases created by argon, germanium, and krypton isotopes. For example, the most abundant Se-isotope ^80^Se cannot be used to measure trace Se concentrations because of the severe interference with argon-dimers, also *m/z* 80, that are also formed in the inductively-coupled plasma (Ralston et al., 2008; Pettine et al., 2015). Moreover, organic carbon and high sodium concentrations can non-specifically affect the Se-signal. Due to the high ionization potential of Se, the addition of organic carbon compounds such as methanol, ethanol, or propanol can enhance the ICP-MS signal for Se. Therefore, for the ICP-MS analysis of Se, it is essential to work with internal standards and use standardized approved methods. In order to reduce or eliminate polyatomic interference, devices equipped with additional collision/reaction cell (CRC) technology have been introduced. These devices allow the detection of the most abundant Se-isotopes by avoiding the interference with argon from the plasma.

### Organic selenium

**Figure 2:**
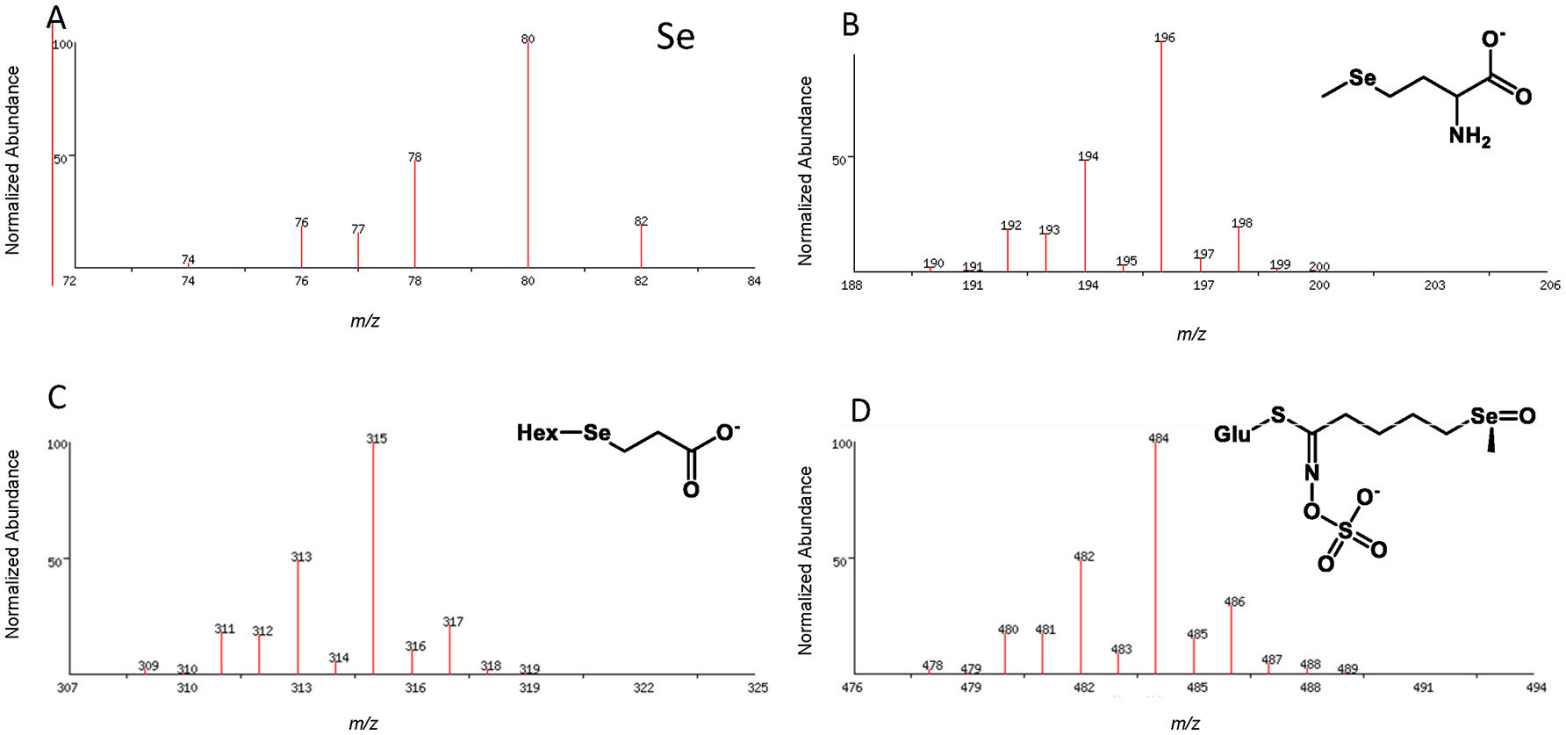
Isotope pattern for elemental selenium **(A)** and as observed in the pseudomolecular ion (M-H)^−^ for organoselenium species, e.g., selenomethionine selenosugar derivative **(B)**, Deamino-selenocysteine-selenosugar **(C)**, Se-glucoraphanin **(D)**; Hex, hexose; Glu, glucose.

*In planta*, Se is involved in biochemical pathways that are analogous to S, resulting in the potential production of a large number of Se-containing metabolites with widely different physical properties which make their analysis a complex task. Different strategies need to be applied to identify and quantify these chemically diverse Se-species. Most frequently chromatographic separation methods are used for the physical separation of volatile or non-volatile Se-metabolites which are then detected, identified and measured by mass spectrometry (MS). The relatively large mass deficiency, coupled with a distinctive isotope pattern (Figure 2), and the high atomic mass relative to carbon and oxygen, greatly facilitates the detection, assignment of molecular formulae and identification of Se-containing compounds by high resolution mass spectrometry. The chromatographic methods include high performance liquid chromatography (HPLC or UHPLC), gas chromatography (GC), capillary electrophoresis (CE), and gel electrophoresis. For HPLC separations, reversed phase chromatography, ion pair chromatography, ion exchange chromatography, and size exclusion chromatography have all been employed (e.g., summarized in Lobinski et al., 2000; Uden, 2002). Among many others, anion exchange (Pedrero et al., 2007) and reversed phase column chromatography (McKenzie et al., 2009; Peñas et al., 2012) have been used for the separation of selenate, selenite, SeMet, SeMeCys, SeCys and other metabolites. More recently hydrophilic interaction liquid chromatography (HILIC) chromatography has been explored as method for analysis of amino acids and selenosugars (Aureli et al., 2012).

### Selenoglucosinolates

**Figure 3:**
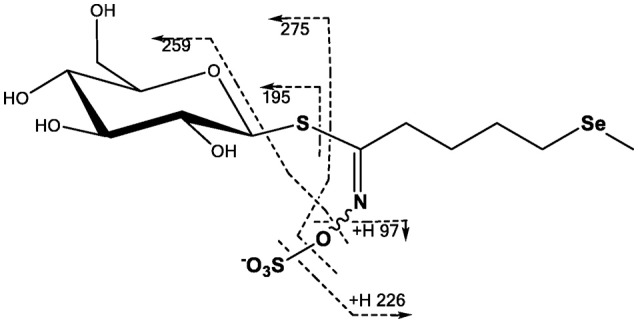
Proposed MS/MS fragmentation pathway for glucoselenoerucin based on fragmentation analysis of glucosinolates (Cataldi et al., 2010; Lelario et al., 2012; Matich et al., 2012).

*Brassica* glucosinolates may contain up to four S atoms. Substitution with Se is possible at any of these sites with the Se entering as SeMet leading to a methylselenide sidechain, as SeCys (proposed for the thioglucose moiety) or directly as selenate. To date mainly incorporation of Se via SeMet has been observed (Matich et al., 2012), but an incorporation via the SeCys into the glucose moiety was also observed (Bertelsen et al., 1988). Glucosinolates may be analyzed by HPLC or LC-MS either directly or after removal of the sulphate group. Se-glucosinolates have been successfully analyzed by LC-MS (Matich et al., 2012, 2015). The location of Se in selenoglucosinates may be determined by LC-MS/MS analysis using well-established negative ion fragmentations (Figure 3). The elemental composition of these fragment ions has been validated using tandem MS and ion trap analysis of the ^32^S and ^34^S isotope distributions in daughter ions derived from the glucosinolate M+2 (^32^S and ^34^S) pseudomolecular ion (Cataldi et al., 2010; Figure 3).

The increasing power of very high mass resolution LC-MS instruments to identify Se-containing metabolites in *Brassicas* has been demonstrated by Ouerdane et al. (2013). Selenosugars, selenosinapine, and selenourea derivatives have also been reported in seed of black mustard (*B. nigra*), grown on naturally Se-rich soil. These identifications are based on high resolution LC-MS, however, the exact structures of these compounds cannot be determined from the mass spectral data alone.

### Selenium-containing volatiles

Incorporation of Se into amino acids, glucosinolates, and other precursors, provides the potential for the formation of novel Se containing volatiles when these precursors are subjected to enzymatic action or non-biotic degradation. Se containing volatiles may arise via three biosynthetic pathways. Firstly direct substitution of Se for S in the amino acids cysteine, SeMeCys and Met leads to the production of dimethylselenide (Me_2_Se), dimethyldiselenide (Me_2_Se_2_) and dimethylthioselenide (MeSSeMe) in transgenic tobacco (*Nicotiana benthamiana*; Matich et al., 2009), Se-enhanced green onions (*Allium fistulosum*; Shah et al., 2007) and *Brassicas* (Matich et al., 2012; Ouerdane et al., 2013). Enzymatic degradation of MeSeCys or SeMet may also result in the production of 2-(methylseleno)acetaldehyde, or the methylselenides so produced may react further with unsaturated aldehydes (2-alkenals) resulting from lipid oxidation (Matich et al., 2009). Thirdly, enzymatic hydrolysis of methylselenoalkyl-glucosinolates leads to methylselenoalkylnitriles and methylselenoalkylisothiocyanates (Matich et al., 2012, 2015).

Many Se-containing volatiles can be readily analyzed by standard GC-MS methods as used for their S-containing analogs. Gas chromatography has been used to separate Me_2_Se and Me_2_Se_2_ and others (Kubachka et al., 2007) as well as Se-containing glucosinolate breakdown products (Matich et al., 2012, 2015). However, selenoxides (e.g., dimethylselenoxide Me_2_SeO analogous to dimethylsulphoxide Me_2_SO) containing beta hydrogens (R_2_CHC(SeO**)**R_2_) and selenones (analogous to sulphones such as dimethylsulphone Me_2_SO_2_) are thermally unstable above room temperatures and are not suitable for standard GC-MS analysis. For highly volatile selenides such as Me_2_Se and Me_2_Se_2_, headspace analysis using highly adsorbent graphite based SPME phases such as Carboxen^TM^ or Carboxen^TM^-PDMS hybrid fibers (Matich et al., 2009) should be preferred. Further increases in sensitivity could be expected by the application of Stir Bar Sorptive Extraction SBSE (Twister) combined with cyrofocusing of volatiles onto the GC column.

### New methods for discovery of selenometabolites

The finding and measurement of trace amounts of novel Se compounds in complex plant extracts can be difficult and tedious. Chromatographic separations coupled with ICP-MS reliably identify HPLC fractions containing Se but do not provide the molecular mass or information about the structure of the Se-containing molecules. This identification relies on the comparison of retention times with authentic reference compounds. Electrospray ionization mass spectrometry (ESI-MS) is very useful for the identification of Se-containing metabolites, however finding minor Se containing species in complex plant extracts is tedious and has disadvantages such as the oxidation of small Se-molecules and lower sensitivity engendered by the complex isotope distribution of Se. A robust approach, which should be also applied for the analysis of *Brassica* vegetables, is the combination of ICP-MS and ESI-MS. Such an approach has been used to investigate Se metabolites in kale (Chan et al., 2010).

Alternatively, bioinformatics approaches, based on the mass defect and Se isotopic ratios, may be used to identify Se-containing metabolites in complex plant extracts. Such strategies are susceptible to automation and the mass defect approach has been used to find Se-containing volatiles in Se-enriched green onions (*A. fistulosum*; Shah et al., 2007). The alternative would be to use the Se isotope pattern as a search requirement for the identification of putative Se-containing metabolites. Such an approach, based on relative isotope abundance and ultra-high resolution FT mass spectrometry, has been implemented to identify all S-containing metabolites in *Allium* species (Nakabayashi et al., 2013) but has not yet been applied to Se-containing metabolites. The application of such analytical approaches will help elucidate the principles that govern relationships between biological metabolites in brassicaceous vegetables and provide important tools for gaining deeper insight into Se metabolism in plants and humans.

## Selenoglucosinolates for human nutrition

### Bioavailability and metabolism of selenoglucosinolates

Selenium is an essential micronutrient for humans, and is part of the 21st amino acid, SeCys, and therefore of selenoproteins. In most Se-dependent enzymes, SeCys is part of the active site, and Se often functions as a redox center in these enzymes. An detailed overview about the Se metabolism in humans is given by Roman et al. (2014). The bioavailability of Se strongly depends on the chemical form in the food. In plants a multitude of different species have been identified such as selenate, selenite, selenocystine, SeMet, selenohomocysteine, MeSeCys, γ-glutamyl-selenocystathionine, SeMet selenoxide, γ-glutamyl-MeSeCys, selenocysteineselenic acid, Se-proponylselenocysteine selenoxide, Se-methylselenomethionine, selenocystathionine, Me_2_Se_2_, selenosinigrin, and other selenoglucosinolates, selenopeptides and selenowax (Navarro-Alarcon and Cabrera-Vique, 2008). In animal tissues selenocompounds are SeCys, SeMet, selenotrisulphides of cysteine, selenosugars, selenite, and selenate.

In humans, Se is mainly ingested and absorbed as SeMet, but also as selenate and selenite. The absorption efficiency of those compounds is supposed to range between 80 and 90% (Patterson et al., 1993; FAO/WHO, 2002). For all other Se-compounds, and especially for selenoglucosinolates, no studies on bioavailability have been systematically conducted so far. With regard to bioavailablility, the limiting step is not the absorption of Se but rather its conversion into metabolically active forms. In general, the human body metabolizes the various Se forms into hydrogen selenide (H_2_Se). H_2_Se is the key metabolite formed from inorganic sodium selenite via selenodiglutathione through reduction by thiols and NADPH-dependent reductases and released from SeCys by a lyase-dependent reaction (Bjornstedt et al., 1992). H_2_Se provides Se for the synthesis of selenoproteins (Ganther, 1999, Figure 4). Also at this stage, it is unclear if and how selenoglucosinolates could be metabolized to release Se for selenoprotein synthesis. Alternatively, they could exert their effects only directly without modulating selenoprotein expression. In addition, their metabolism has not been studied yet.

**Figure 4:**
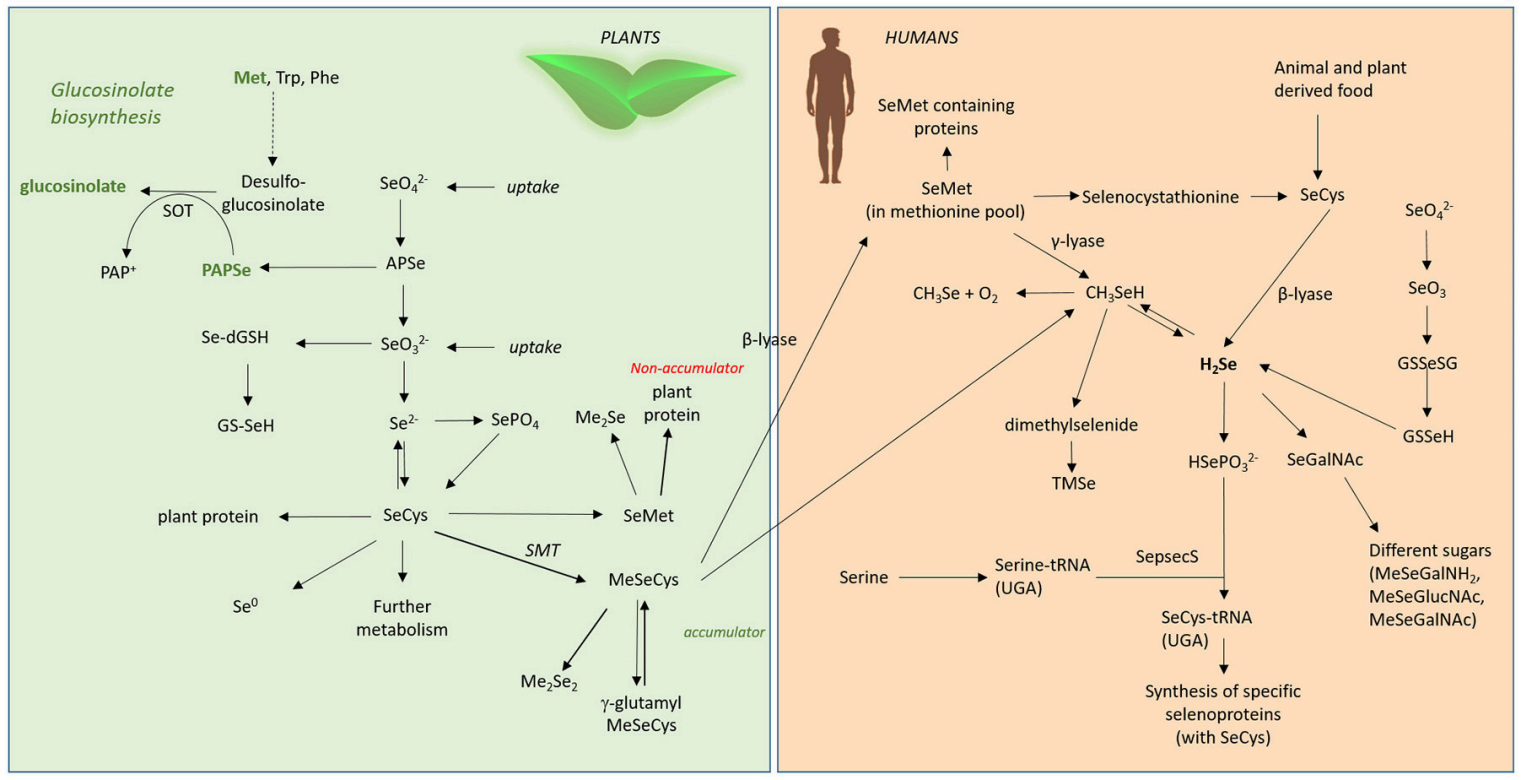
Se metabolism in plants and humans. Met, methionine; Trp, tryptophane; Phe, phenylalanine; SOT, sulphotransferase; ${\text{SeO}}_{4}^{2-}$, selenate; ${\text{SeO}}_{3}^{2-}$, selenite; APSe, Adenosine-5′-phospho-selenate; Se-dGSH, seleno-diglutathione; GS-SeH, glutathioselenol; SePO_4_, selenophosphate; SeCys, selenocysteine; SMT, selenocysteinemethyltransferase; SeMet, selenomethionine; MeSeCys, methylselenocysteine; Me_2_Se, dimethylselenide; Me_2_Se_2_, dimethyldiselenide; γ-glutamyl-MeSeCys, γ-glutamyl-methylselenocysteine; H_2_Se, hydrogen selenide; CH_3_Se, methylselanyl; CH_3_SeH, methylselenol; SeGalNAc, seleno N-acetylgalactosamine; SepsecS, Sep (O-phosphoserine) tRNA:Sec (selenocysteine) tRNA synthase; ${\text{HSePO}}_{3}^{2-}$, selenophosphate; TMSe, trimethylselenonium.

It is also important to consider that many brassicaceous vegetables are processed by chopping, cooking or freezing. Such processing has been shown to severely influence the glucosinolate profile (Hanschen et al., 2014), and would also be expected to modulate the selenoglucosinolates within the vegetable matrix. To provide knowledge about the amount of their corresponding health-promoting breakdown products, the effect of various processing procedures on the degradation of selenoglucosinolates must be considered as well.

### Chemo-preventive effects of selenoglucosinolate related products

Selenium, often labeled as “antioxidant,” is actually not an antioxidant compound by itself, but rather an essential part of the catalytic center of selenoproteins, which are involved in protection against oxidative stress (Steinbrenner and Sies, 2009). Among the selenoproteins there are well-known redox-active selenoenzymes, such as glutathione peroxidase (GPx), thioredoxin reductase (TrxR), and methionine sulphoxide reductase B (MsrB). GPx isoenzymes reduce hydrogen peroxide (H_2_O_2_), organic hydroperoxides, and phospholipid hydroperoxides (only GPx4) using reduced glutathione as co-substrate (Papp et al., 2007; Brigelius-Flohe and Maiorino, 2013). TrxR isoenzymes reduce a wide variety of substrates, including oxidized thioredoxins, H_2_O_2_ and organic hydroperoxides (Bjornstedt et al., 1995), MsrB reduces free and protein-bound methionine sulphoxide to methionine (Moskovitz et al., 2002). Links between sulphur-containing isothiocyanates and selenoprotein production have been described in Barrera et al. (2012). Se in combination with isothiocyanates increased the expression of TrxR1 and GPx2 in colonic cell lines more strongly than Se or isothiocyanates alone (Barrera, 2010). In mice, the combination of the isothiocyanate sulphoraphane and a super-nutritional Se supply was most efficient in upregulating TrxR1 and glutathione-S-transferase activity in the colon (Krehl et al., 2012; see Section Selenoglucosinolates). A similar effect was observed in endothelial cells lines (Campbell et al., 2007) and in a colonic cell line (Wang et al., 2015).

Any excess supply of Se results in increased metabolism, but marginal or no further increases in selenoprotein biosynthesis. Many of the metabolites including H_2_Se and monomethylselenol are highly redox active and generate reactive oxygen species (ROS) upon reaction with and oxidation of thiols. These compounds are therefore termed as redox-active Se compounds (e.g., selenite, selenocystine, methylseleninic acid, MeSeCys that are known to exert oxidative stress; Spallholz, 1994). Such pro-oxidative properties reflect the opposite spectrum of a common consensus that Se is just an antioxidant (Jukes, 1983). Based on this, selenoglucosinolate related compounds were analyzed for their putative redox-modulatory properties: Preliminary studies focused on the effects of artificial phenylalkyl isoselenocyanates. In cell culture, these compounds were shown to be more cytotoxic compared to natural phenylalkyl isothiocyanates, to induce more apoptosis, and to inhibit cell proliferation of human melanoma cells more strongly (Sharma et al., 2008). Further, in a melanoma mouse model they reduced tumor size more efficiently than S-containing analogs (Sharma et al., 2008). The higher anticancer activity of isoselenocyanates was linked to their faster reaction with thiols such as glutathione and their more efficient modulation of the cellular redox status compared to isothiocyanates (Crampsie et al., 2012). Moreover, these artificial compounds effectively decrease Akt-3 signaling in mouse melanoma cells (Sharma et al., 2009) as well as in different xenograft models (Nguyen et al., 2011). Prostate apoptosis protein-4 can further enhance the antitumor activity of the phenylbutyl isoselenocyanate (ISC-4; Sharma et al., 2011) and a synergistic interaction of ISC-4 with the tumor therapeutic agent cetuximab was reported for colon cancer cells and a related xenograft model (Allen et al., 2013). The artificial 4-(methylsulphinyl)butyl isoselenocyanate (ISC-SFN) showed stronger induction of the redox-sensitive transcription factor Nrf2 compared to the corresponding isothiocyanate sulphoraphane (SFN; Emmert et al., 2010). Moreover, ISC-SFN was more cytotoxic to malignant cells but less toxic to non-cancer cells compared to SFN (Emmert et al., 2010). Recently, it was shown that derivatization of ISC-SFN with organofluorine substitutes can further enhance the selective toxicity toward tumor cells (Cierpial et al., 2016). Thus, selenoglucosinolates as possible precursors for related compounds studied in *in vitro* and xenograft models might have a relevant anti-cancer potential, which should be further analyzed in the future.

### Selenoglucosinolate related compounds and their effects on the immune system

Dietary Se plays an important role in inflammation and immunity. Selenium deficiency can lead to significant impairment of immune function and an increased susceptibility to infection and chronic disease (Calder and Kew, 2002). Our current knowledge suggests the effects of Se on the immune system are predominantly mediated through Se incorporation into selenoproteins (Huang et al., 2012). These selenoproteins can initiate or enhance immunity and some are involved in immune regulation.

Se enrichment of the diet is a subject of considerable debate. There is good evidence that supplementation between 100 and 200 μg day^−1^ can be beneficial to immune function, e.g., enhanced cellular immune response and restored age-related decline in immune response in elderly patients (reviewed in Rayman et al., 2008). A study looking at Se supplementation in prawns showed increased phagocytic activity and increased respiratory burst, whilst also inducing a range of antioxidant selenoproteins (Chiu et al., 2010). This highlights the interesting paradox of Se regulating oxidation status (redox tone) in either direction e.g., reducing oxidation status or triggering oxidation. Selenylation of plant polysaccharides, known to be immune-stimulatory, were found to have enhanced immune activity *in vitro* (peripheral lymphocytes) and *in vivo* (chickens) compared to their un-selenated forms (Li et al., 2016), whilst Se-enriched *Lactobacillus brevis* induced interferon γ and interleukin 17 secretion and increased natural killer cell activity in mice compared to *L. brevis* alone (Yazdi et al., 2012). Both studies indicate the Se predominate effect may be immune enhancement rather than regulation.

**Figure 5:**
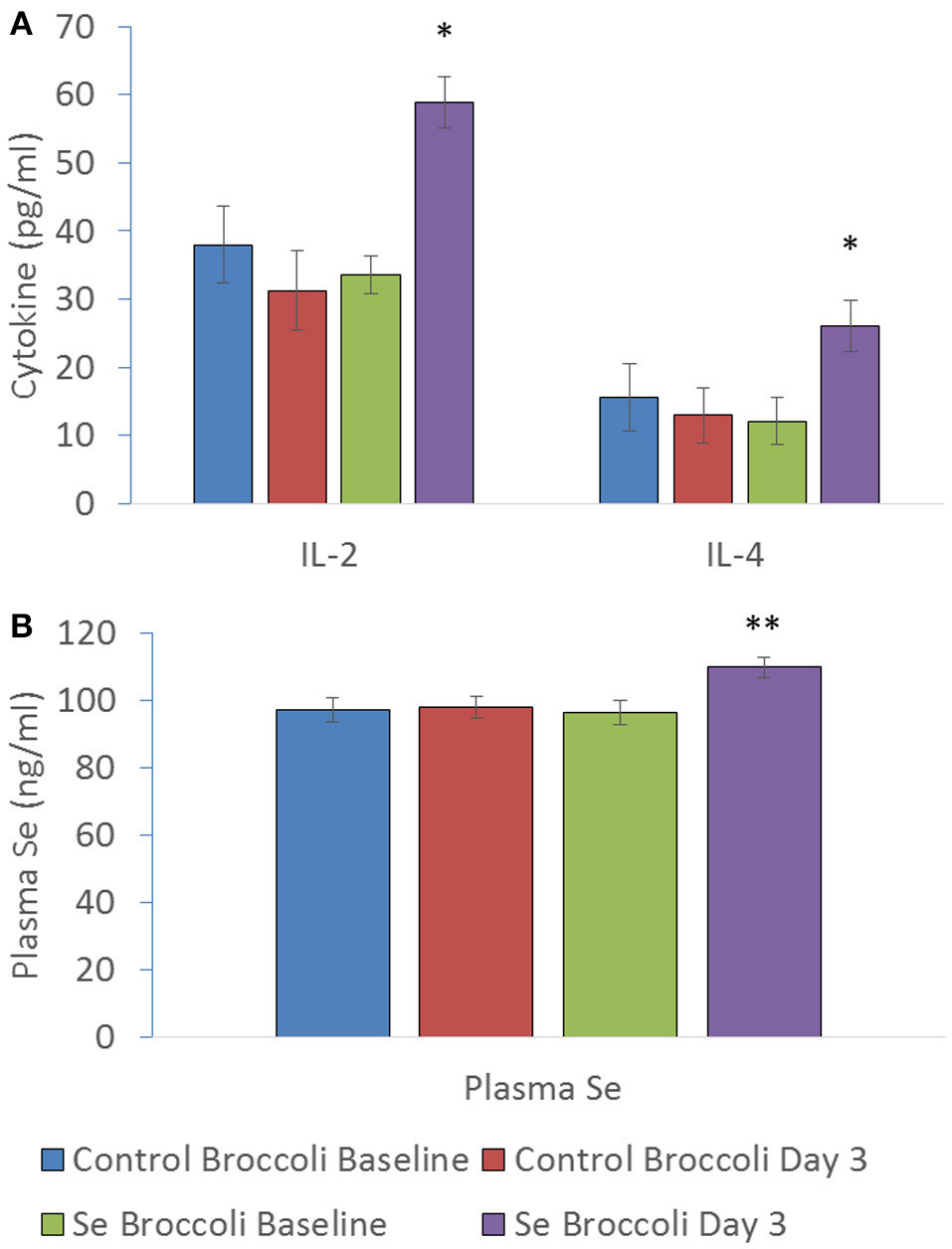
**(A)** IL-2 (pg/ml), IL-4 (pg/ml) and **(B)** plasma selenium (ng/ml) at baseline of control broccoli feeding (C0), end of 3 days of control broccoli feeding (C3), baseline of Se-enriched broccoli feeding (S0) and end of 3 days of Se-enriched broccoli feeding (S3). Results are shown as mean (*n* = 18) ± SE and significance of change between differences seen in both weeks ((S3-S0)-(C3-C0)) are indicated by ^*^p < 0.05 and ^**^p < 0.001 (reformatted from Bentley-Hewitt et al., 2014).

Substantial evidence exists that both Se and *Brassica* compounds, mainly isothiocyanates, impact the immune response through mechanisms predominantly involving oxidation status (redox tone). There does appear to be some opposing effects of Se and isothiocyanates. The former is more commonly related to immune enhancing effects (Hoffmann and Berry, 2008) whilst the latter is linked to downregulation of immune signals (Wagner et al., 2013). Research investigating the effects of Se-enriched broccoli on immune response produced by peripheral blood mononuclear cells challenged *ex vivo* indicated that the overriding effect was immune-stimulatory (Bentley-Hewitt et al., 2014). This study involved participants consuming one serving of control broccoli or Se-enriched broccoli (200 μg Se) for 3 days with a wash-out period between dietary interventions. Plasma Se significantly increased from a baseline of 96 ± 4 ng ml^−1^ to 110 ± 3 ng ml^−1^ after Se-enriched broccoli consumption, along with cytokines interleukin-2 and interleukin-4 production from participants' peripheral blood mononuclear cells when stimulated with phorbol 12-myristate 13-acetate and ionomycin, whilst no increases were observed following consumption of control broccoli (Figure 5). Additionally Se-enriched radish sprouts were found to be immune-stimulatory in hens (Hossain et al., 2010). In contrast, a study testing Se-enriched sauerkraut extracts on a macrophage cell line *in vitro* showed anti-inflammatory effects (Peñas et al., 2012), highlighting a major discrepancy between *in vivo* and *in vitro* studies. It appears that the anti-inflammatory effects of *Brassica* phytochemicals may predominate *in vitro*. This does not explain why Se supplementation of *Brassicas* in Peñas et al. (2012) have enhanced anti-inflammatory activity compared to the same *Brassica* extracts that were not supplemented with Se. However, it suggests that digestion, absorption, Se status, modification of the bioactive compounds by other cell types and/or the complexity of cellular cross talk *in vivo* may have a great influence on how immune cells respond to the bioactive compounds in Se-enriched *Brassicas*. The *in vitro* study by Peñas et al. (2012) includes an extraction procedure in an attempt to isolate the glucosinolate hydrolysis products for testing on a macrophage cell line. Therefore, it is possible that alternative seleno compounds that were removed in the process may drive the immuno-stimulatory effect *in vivo* e.g., seleno amino acids. To start to elucidate the reasons for the discrepancy between *in vitro* and *in vivo* results, one could use *in vitro* digestion of Se-enriched *Brassica* prior to exposure to cells *in vitro* and extract material to include seleno amino acids. Previous research, utilized *in vitro* digestion of Se-enriched broccoli before testing with colon cancer cells and found a reduction in H_2_O_2_ production, however no inflammatory markers were measured (Tsai et al., 2013). At present, information into the effects of Se-enriched *Brassicas* on immune respones particulary human *in vivo* data is limited.

We still do not know the impact of all the Se-containing bioactives, which may be driving the immune response. Research should focus on whether naturally occuring selenoglucosinolate hydrolysis products and amino acids are more bioactive than the S-containing analogs. Additionally, research is required to ascertain whether modification to immune signals, such as increased levels of cytokines, results in a more robust immune response in humans.

## Conclusion/future view

Selenium deficiency or suboptimal Se intake is still regarded as a major health problem for about one billion people worldwide, while an even larger number may consume less Se than required for optimal protection against cancer, cardiovascular diseases, and severe infectious diseases (Haug et al., 2007). Furthermore, due to climate change and climate-soil-interactions, a global Se soil loss of about 8% is predicted by 2099 (Jones et al., 2017). These Se losses will have a higher impact on human health than predicted because Se losses for cropland and pasture are predicted to be 66 and 61%, respectively. These Se losses would be expected to increase global Se deficiency in humans further since the main Se sources for humans are plants and livestock grown on this land.

Due to their distinct biodiversity, *Brassica* vegetables are consumed regularly worldwide. Thus, Se-biofortification of *Brassica* crops is an important biotechnological tool that can be used to benefit of Se nutrition in humans. Furthermore, beside the general Se-metabolites, such as seleno-proteins and seleno aminoacids, which can be found in most plant species, Brassicales also contain specialized Se-containing compounds with health benefiting properties such as MeSeCys. Also, the recent discovery of significant amounts of the selenoglucosinolates in biofortified broccoli is encouraging as it opens a further avenue for the production of potentially health promoting compounds in this genera. However, so far no investigations have been conducted on the biosynthesis of selenoglucosinolates or on their bioefficacy in human health, which would be of particular interest due to the distinct protective potential of their potential hydrolysis products.

Increasing Se uptake by the Brassicales is best achieved via hydroponic means, where Se exposure can be carefully controlled and uptake maximized. For crops already grown using hydroponic or similar systems, such as drip lines, Se biofortification should be relatively easy to implement. However, alongside this it will be critical to consider issues relating to Se toxicity, as even Brassicales of the same species can have variable Se uptake rates. This means that a “one size fits all” approach cannot be implemented and biofortificaiton regimes will have to be established for each crop and over the entire plant growth period in order to produce material with a known and stable Se content. Despite this, Se fertilization strategies have been successfully achieved in Finland to counter Se deficiency and should be investigated by other countries where the malnutrition of Se effects the human health of their population.

Although, critical Se intake levels have already been determined with respect to Se undernourishment, we still need to understand the modes of action of individual Se-compounds in human metabolism before recommendations can be made for specific diseases. So far, no daily intake recommendation is established regarding the prevention of chronic diseases such as cancer or the maintenance of a well-regulated immune system. Moreover, further investigations are required to better understand the bioavailability and molecular effects of the selenoglucosinolates before determining their effective concentration for protection against chronic diseases. Achieving these goals will further establish the role Se plays in supporting human health, particularly through members of the Brassicales.

## Author contributions

MW: Corresponding author; MS, FSH, DS, MM: Section Selenium in Brassicales; SB and DR: Section Instrumental Approaches for Detecting, Measuring, and Monitoring Selenium and Its Metabolites within Brassica Species; AK, KBH, FSH, MW: Section Selenoglucosiolates for Human Nutrition.

### Conflict of interest statement

The authors declare that the research was conducted in the absence of any commercial or financial relationships that could be construed as a potential conflict of interest.

## Footnotes

^1^https://ods.od.nih.gov/factsheets/Selenium-HealthProfessional/
